# Impact of health shocks on household consumption structure

**DOI:** 10.3389/fpubh.2024.1431833

**Published:** 2024-07-08

**Authors:** Yinxin Qiu, Fen Zhang

**Affiliations:** Economics and Management School, Wuhan University, Wuhan, Hubei, China

**Keywords:** health shocks, household consumption, medical expenditures, health, consumption structure

## Abstract

**Introduction:**

In the aftermath of the pandemic, the impact of health shocks on household expenditure patterns has become a critical area of focus due to the heightened uncertainty surrounding consumers’ expectations. Household medical expenditures have emerged as a key factor in the evolving consumption structure.

**Method:**

This research developed a practical framework to assess the influence of health shocks on family spending patterns, focusing on health shortfalls. Health emergencies were measured through randomized medical spending. Data was sourced from the 2010-2018 Wave 5 Statistical Report of the China Family Panel Studies (CFPS), which included a total of 25,809 participants.

**Results:**

The findings revealed that health shocks significantly increased the proportion of household spending on medical expenses. Concurrently, sub-expenditures such as food and education were reduced to varying degrees as households adjusted their consumption patterns to mitigate the impact of health shocks. The effects of these shocks were more pronounced in low-income households and those with health insurance.

**Discussions:**

The government should take steps to promote public health, reduce the burden of medical expenses resulting from health shocks, and unlock the consumption potential. Additionally, efforts should be made to boost economic growth and systematically upgrade household consumption patterns to effectively cushion the impact of health shocks.

## 1 Introduction

At a particular stage of economic development, consumption becomes a crucial driver for long-term economic growth. It promotes sustainable development, stimulates economic growth, and improves residents’ welfare. According to the National Bureau of Statistics of China, in 2023, final consumption expenditure, gross fixed capital formation, and net exports of goods and services are expected to drive economic growth by 4.3, 1.5%, and −0.6%, respectively, contributing 82.5, 28.9%, and −11.4% to economic growth. However, despite being a primary driver, China’s consumption remains at lower levels compared to countries at a similar stage of development globally. In response, China has placed a strong emphasis on promoting consumption from 2023 to 2024.

The adverse impact of health shocks, such as COVID-19, has significantly affected individual consumption choices in recent years. Consequently, medical expenditure has become a new focus in the study of Chinese household consumption structures. The Central Economic Work Conference in 2023 emphasized the need to stimulate consumption potential. Therefore, examining the latest changes in Chinese household consumption patterns holds considerable practical significance.

Recent research has highlighted that several factors, such as household disposable income and savings, play a significant role in shaping household consumption structures. Additionally, uncertainty influences household spending patterns, prompting consumers to adopt precautionary saving behaviors and encounter liquidity constraints (1–3). With advancements in health economic theories, specific studies have delved into how health shocks impact household spending patterns (4–6). Given the ongoing challenges in global economic recovery and the uncertain future predictions for consumers, it becomes crucial to examine the multidimensional impact of health shocks on household consumption patterns. Published studies have employed various methods to assess health shocks, including self-reported health conditions (7), the number of days unable to carry out daily activities due to illness (8), and recent hospitalizations (4). This paper introduces innovation by utilizing stochastic medical expenditure (the logarithmic of the difference between actual and predicted medical expenditures) to measure health shocks, thereby providing a more accurate measure of health shocks.

The rest of the paper is structured as follows: Section 2 presents an overview of the previous literature on health shocks and consumption. Section 3 describes the data sources and variables used in our analysis. Section 4 empirically analyses the impact of health shocks on household consumption across various tiers. Finally, the last section presents the conclusions, limitations, and suggestions.

## 2 Literature review

“Consumption” refers to the expenditure on goods and services to fulfill individuals’ needs. Various external factors, including disposable income, expenses, and education, significantly affect household purchasing strategies. Liu et al. (9) have proposed that expenditure uncertainty results in prudent household savings, further restricting the liquidity of consumers. Conversely, credit markets can smooth out the detrimental impact of uncertain shocks and alleviate the liquidity constraints of residents (10). Song et al. (5) have demonstrated that health shocks significantly increase out-of-pocket medical expenditures in the short term, with a continuous decline in total household consumption.

Most health economic theories, such as the health deficit model developed by Strulik (11), suggest that consumers optimize their consumption over time to maximize utility. Nevertheless, such theories are frequently criticized for their overly idealistic assumptions. The challenge has prompted scholars to pay greater attention to the element of uncertainty. For instance, Frini (12) has empirically checked the effect of unemployment on aging-saving link. Bande et al. (13) have found the evidence that a precautionary motive for saving when uncertainty was proxied by the unemployment rate. Lugilde et al. (14) have confirmed the impact of uncertainty on residential consumption based on these hypotheses above. Presently, uncertainty is widely regarded as a significant explanatory variable of consumption theories.

Scholars in China have begun studying the impact of uncertainty on the consumption strategies of Chinese residents, employing theoretical models such as the precautionary saving model. Tan (3) found that residents in rural areas tend to reduce current spending and increase savings in response to uncertain futures. Wang (15) demonstrated that consumption uncertainty has a more pronounced adverse effect on farmers’ consumption demand. Chinese scholars have utilized precise data to calculate indicators of uncertainty, such as the ratio of different expenses to disposable income (16, 17). Tan (3) employed a modified deviation rate to assess the uncertainty of income and expenses. Additionally, Xu et al. (18) used the psychological deviation rate to quantify consumer uncertainty.

With the rise of health economics, economic analysis has begun to focus on the impact of health shocks on the household consumption structure in the context of uncertainty. Moreover, during the COVID-19 pandemic, the epidemic as a health shock has thoroughly influenced consumption levels and patterns both domestically and internationally (19). As a result, academics have shown growing interest in this matter. Health shocks can directly affect overall household spending, resulting in a reallocation of consumption among various categories. Liu et al. (4) have demonstrated that health shocks in rural households lead to a notable increase in overall household spending and the proportion of medical expenses, alongside a significant decrease in the share of various consumption sub-categories. Song (5) and Shi (20) have discovered a decrease in overall household consumption, with a particular reduction in food intake to lessen the adverse effects of health shock. Chen et al. (21) analyzed data on COVID-19 cases in China. They found that the pandemic has significantly influenced Chinese household consumption, with the most profound decline in demand for food, entertainment, and tourism.

Concerning the consumption strategies of Chinese households, Zheng and Chen (22) have employed the CHNS dataset to demonstrate that health shocks influence residents’ food and health expenditure. Nevertheless, the urban basic medical insurance has significantly mitigated such fluctuations. Based on CHIP research, Tu et al. (23) found that rigid expenditure induced by health shocks reduces agricultural inputs among farmer households, leading to decreased production efficiency and thus intensifying economic vulnerability.

Due to the ongoing decline in household spending and the lack of upgrading in China’s consumption structure, some domestic studies have focused on the impact of health shocks on household consumption. However, these studies have not sufficiently accounted for the impact of uncertainty when selecting proxy variables for health shocks. This paper proposes a suitable approach to evaluate the impact of health shocks on household expenditures. The method is based on the uncertainty estimation technique developed by Luo (24) and uses random medical expenditure as a proxy variable for health shocks, making it more aligned with the measure of health shocks. Furthermore, this paper examines the heterogeneity of the samples by considering factors such as age, income, gender, and health insurance to obtain more reliable and comprehensive findings.

## 3 Data sources and variable descriptions

This paper referred to the health economic theories of Grossman (25) and Dalgaard (26) to construct an empirical model. Given the panel nature of the data, we employ Least squares dummy variable method (LSDV) to control year fixed effects. The regression equation is shown below.


$$Y_{\mathrm{it}}=\theta_{i}+\beta Z_{\mathrm{it}}+\delta \sum X_{\mathrm{it}}+\sigma_{\mathrm{it}}$$

Considering the presence of omitted and time-independent variables in the household sample, the fixed-effects model was employed following the Hausman test. $\theta_{t}$ is a time-fixed effect in a period t , while $\sigma_{\mathrm{it}}$ represents a random perturbation term. Variable definitions were derived from the CFPS questionnaire, with relevant descriptions provided in Table 1. The construction of some of the variables is described below:

**Table 1:** Variable definition.

	Variable identifier	Variable description
Key variables	Health shock	Stochastic medical expenditures, the logarithmic value of the difference between actual and projected medical expenditures
	Percentage of medical expenditures (MedExpend %)	The proportion of household expenditure allocated to medical costs
	Percentage of food expenditure(FoodExpend %)	The proportion of household expenditure allocated to food costs
	Percentage of Recreation Expenditure (EecExpend %)	The proportion of household expenditure allocated to cultural, educational, and recreational programs
	Percentage of welfare expenditure(EpwelfExpend %)	The proportion of household expenditure allocated to improving the quality of life and the economic situation
Individual characteristic variables	Age	The age range of the sample individuals was between 18 and 60 years old
	Gender	Male = 1, female = 0
	Marriage	The categories are as follows: 1 for unmarried, 2 for married with a spouse, 3 for cohabiting, 4 for divorced, and 5 for widowed.
	Weekly working hours	The number of hours worked per week
	Education	The level of educational levels from 0 to 10
	Head of household	Head of household = 1, not = 0
	health status	Composite health index constructed with values ranging from 0–5.66
	Health insurance	Purchasing health insurance = 1, not = 0
	Oopshare	The proportion of out-of-pocket medical expenses.
Household characteristics variables	Family size	Measuring household size by the number of people in the household
	lnfincome	The logarithm of the amount of *per capita* household income
	lnasset	The logarithm of the net worth level of the household
Regional characteristic variables	Urban	Located in a city = 1, located in a village = 0
	Regional pollution	The presence of highly polluting firms within 5 kilometers, with a value of 1 for presence and 0 for absence.
	Regional economy	For observations of regional economic conditions, a minimum value of 1 indicates very poor, and a maximum value of 7 indicates very rich.

$Y_{\mathrm{it}}$ is the dependent variable indicating the proportion of a given household consumption category at time t. This variable was employed to evaluate the household’s consumption structure. The National Bureau of Statistics (NBS) has established a classification system for household consumption strategies. It divided expenditure into several categories: food, clothing, housing, living goods and services, transport and communication, education and entertainment, healthcare, and other goods and services (4). Our study focused on the consumption categories of medical, food, recreation, and welfare expenditures. Alterations in the ratios of these categories indicated changes in household consumption structure.

$Z_{\mathrm{it}}$ represents the core independent variable utilized to construct and calculate the health shocks indicator. Following the estimation method of Luo (24), we selected stochastic medical expenditure to gauge the uncertainty of household medical expenditures, which is the logarithmic value of the difference between predicted and actual medical expenditure. The logarithmic value of household medical expenditures was regarded as the dependent variable. Age structure, health insurance, health condition, and income level were utilized as independent variables to estimate the function of household medical expenditures. In the process of estimating the medical expense function, we used the Sample Selection Model for testing. With the significant inverse Mills ratio coefficient, we estimated the medical expense function using the Heckman model. The stochastic medical expenditures were then obtained by subtracting the predicted medical expenditures from the actual medical expenditures. In the subsequent robustness tests, whether an individual has had health issues in the last two weeks was selected as an alternative health shock proxy.

$X_{\mathrm{it}}$ is a control variable. It contains a series of variables that may affect the dependent variables, including, but not limited to, age, income, health level, and health insurance purchase status. This document utilized Blundell’s comprehensive health status measurement methodology, which merges subjective and objective health indicators. Employing the CFPS database and derived from the initial questionnaire structure, we developed a comprehensive indicator of health condition using a range of daily living activity metrics (ADLs), including the ability to go outside and eat independently. This measurement, fluctuating continuously between 0 and 5.66, was used in subsequent empirical studies.

Based on the available literature, our study controlled for personal, household, and regional factors. Personal variables included in the analysis were age, age squared, gender, marital condition, weekly working hours, education, head of the household, the proportion of out-of-pocket medical expenditures, and health insurance. Household variables included household size, the logarithm of household income per person, and the household net worth. Regional factors include household location, pollution status, and regional economic environment.

The study data was derived from the China Family Panel Studies (CFPS), which encompasses necessary indicators, such as health status and household consumption structure. We utilized cross-sectional data from the baseline study 2010 to create an unbalanced panel dataset covering five intervals up to 2018. The empirical data focused on the age group of 18 to 60. The data cleansing procedure omitted specific samples without information on critical variables. Ultimately, 92,952 observation data remained, involving 25,809 participants.

**Figure 1:**
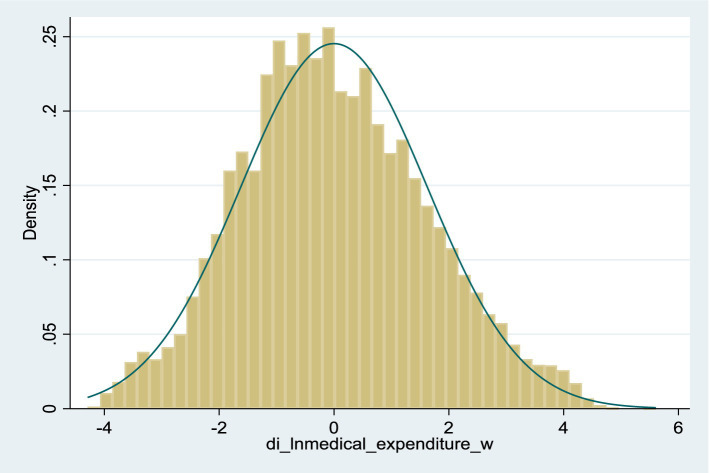
Histogram of health shock distribution.

Descriptive statistics for selected variables’ mean, standard deviation, maximum, and minimum values are presented in Table 2. The analysis revealed that food expenditure constituted a notably more significant fraction of overall consumption than the other three categories. The health shock indicator is measured by the logarithmic difference between the actual value and the theoretical value of medical expenditures, with an average close to zero. Figure 1 shows the distribution of health shocks.

**Table 2:** Descriptive statistics.

	Mean value	Standard deviation	Minimum value	Maximum value
Health shock	0	1.63	−4.29	5.61
Percentage of medical expenditures	0.09	0.14	0	1
Percentage of food expenditure	0.34	0.2	0	1
Percentage of recreation expenditure	0.09	0.14	0	1
Percentage of welfare expenditure	0.02	0.06	0	1
Health status	2.75	0.68	1.49	4.35
Age	41.11	11.57	18	60
Head of household	0.41	0.49	0	1
Weekly working hours	19.3	30.57	0	168
Education	2.89	1.46	0	7
Health insurance	0.88	0.32	0	1
Income	14,512.79	43,241.27	0	10,299,996
Oopshare	2.95	10.15	0	80.5
Family size	4.34	1.86	1	26
lnfincome	8.97	1.27	−1.61	14.21
lnasset	12.29	1.38	0	17.75
Urban	0.49	0.5	0	1
Regional pollution	4.21	1.6	1	5
Regional economy	4.59	1.39	1	7
Sample size	92,952			

## 4 Results

Our paper has analyzed the effect of health shocks on the consumption structure of households. The empirical results in Table 3 show the specific impact of health shocks on various types of household consumption. The findings indicate that the proportion of household medical spending has markedly increased, accompanied by a noticeable decline in the proportion of food expenditure. The share dedicated to educational and recreational activities changed briefly. The effect of health shock on share of welfare expenditure is significant, but is very small.

**Table 3:** Regression analysis of health shocks on consumption structure.

	MedExpend %	FoodExpend %	EecExpend %	EpwelfExpend %
Health shock	1.823*** (0.0424)	−0.759*** (0.0604)	−0.261*** (0.0443)	−0.030* (0.0174)
Age	−0.459*** (0.0491)	−0.460*** (0.0699)	1.057*** (0.0512)	0.096*** (0.0202)
Age squared	0.007*** (0.0006)	0.006*** (0.0008)	−0.013*** (0.0006)	−0.001*** (0.0002)
Gender	−0.088 (0.1433)	1.019*** (0.2042)	−0.674*** (0.1497)	−0.124** (0.0590)
Marriage	0.358*** (0.1326)	0.518*** (0.1889)	−0.871*** (0.1384)	0.126** (0.0544)
Worktime	−0.007*** (0.0026)	−0.029*** (0.0037)	−0.005* (0.0027)	0.001 (0.0011)
Education	−0.210*** (0.0584)	−0.949*** (0.0833)	1.083*** (0.0611)	0.249*** (0.0241)
Head of household	−0.218 (0.1439)	−0.328 (0.2051)	0.312** (0.1503)	−0.124** (0.0592)
Health insurance	0.378 (0.2324)	−0.277 (0.3312)	−1.265*** (0.2429)	0.203** (0.0956)
Family size	0.244*** (0.0402)	0.002 (0.0574)	0.363*** (0.0421)	−0.030* (0.0165)
lnfincome	−0.560*** (0.0625)	0.305*** (0.0891)	−0.467*** (0.0653)	0.045* (0.0257)
lnasset	−1.230*** (0.0577)	−1.009*** (0.0823)	−0.112* (0.0603)	0.545*** (0.0237)
Urban	−1.128*** (0.1505)	4.069*** (0.2143)	−0.035 (0.1571)	0.369*** (0.0619)
Year fixed effects	Control	Control	Control	Control
Sample size	38,182	38,179	38,147	38,086
R-value squared	0.088	0.074	0.033	0.053
*F*-value	217.740	179.392	76.314	125.933

The year fixed effects are controlled; Standard errors in parentheses; *p < 0.1, **p < 0.05, ***p < 0.01.

Furthermore, Table 3 reveals a significant correlation between most variables and the percentage of different consumption types. With age increasing, there was a declining trend in the share of food and medical expenses, in contrast to a rising trend in the proportion of the expenditure on education and leisure activities. The consumption structure varied based on gender and marital status, with households having spouses allocating more toward food and medical expenses. Similarly, higher income and net assets *per capita* resulted in a lower proportion of expenditure on education and recreation. In order to control for year-specific characteristics, a set of dummy variables were utilized in the model as control variables. The results indicated that all the coefficients are significant, and the year fixed effects are effectively controlled.

## 5 Discussion

The empirical research began with regression analyses to explore the effect of health shocks on the consumption structure. Subsequently, the results were validated through robustness assessments with health shock proxy variables. By stratifying the sample according to age, gender, income, and health insurance acquisition, this paper explored the differential impacts of health shocks on the consumption structure. Current studies on health shocks and consumption structure indicated the varied influence of health shocks on household consumption. Song et al. (5) and Shi et al. (20) believed that household consumption would diminish, primarily focusing on food intake to mitigate health shocks. Liu et al. (4) found that health shocks increase total household consumption and various sub-category expenditures, with the highest increase observed in medical expenditures. Despite these varying perspectives, there is a consensus that health shocks increase the proportion of medical expenditures.

Empirical findings in Table 3 illustrate the distinct effects of various consumption strategies in response to health shocks. The percentage of household medical expenditures has significantly risen, while the proportion of expenditures on food, education and recreation has declined. Generally, with a steep increase in the proportion of medical expenditure, the percentages of different consumption types decreased to various extents. This phenomenon suggests that medical consumption, to some extent, crowds out non-medical consumption, leading to a redistribution of household spending and change in the household consumption structure, which aligns with the theoretical analysis previously discussed.

This paper identified significant illness shocks as markers for adverse health effects, validated by earlier research (4). For analysis, we chose the conventional health shock measures in the survey, specifically whether the participant experienced illness in the last two weeks. Findings in Table 4 reveal that using the proxy marker for health shocks resulted in a significant increase in the share of medical expenditure and a notable reduction in the percentage of food expenditure. The proxy indicator showed insignificant effects on expenditures related to leisure activities and welfare conditions. These results imply that the impact of health shocks on consumption patterns remains consistent, maintaining the primary findings of this research unchanged.

**Table 4:** Robustness test (acute illness within two weeks).

	MedExpend %	FoodExpend %	EecExpend %	EpwelfExpend%
If illness recently	2.788*** (0.1118)	−1.975*** (0.1629)	−0.211* (0.1175)	0.019 (0.0465)
Age	−0.415*** (0.0329)	−0.239*** (0.0480)	0.790*** (0.0346)	0.094*** (0.0137)
Age squared	0.006*** (0.0004)	0.003*** (0.0006)	−0.010*** (0.0004)	−0.001*** (0.0002)
Gender	0.155 (0.1031)	0.726*** (0.1502)	−0.619*** (0.1084)	−0.115*** (0.0429)
Marriage	0.375*** (0.0970)	0.496*** (0.1414)	−1.167*** (0.1019)	0.075* (0.0403)
Worktime	−0.008*** (0.0018)	−0.022*** (0.0027)	−0.013*** (0.0019)	0.001* (0.0008)
Education	−0.138*** (0.0418)	−0.881*** (0.0609)	1.020*** (0.0439)	0.245*** (0.0174)
Head of household	−0.533*** (0.1073)	−0.199 (0.1564)	0.315*** (0.1128)	−0.072 (0.0447)
Health insurance	0.806*** (0.1618)	−1.312*** (0.2358)	−0.867*** (0.1701)	0.292*** (0.0673)
Family size	0.353*** (0.0284)	0.064 (0.0414)	0.192*** (0.0299)	−0.048*** (0.0118)
lnfincome	−0.575*** (0.0451)	0.204*** (0.0658)	−0.491*** (0.0475)	0.041** (0.0188)
lnasset	−0.930*** (0.0416)	−1.134*** (0.0606)	−0.148*** (0.0437)	0.584*** (0.0173)
Urban	−0.872*** (0.1082)	4.236*** (0.1576)	−0.073 (0.1137)	0.379*** (0.0450)
Year fixed effects	Control	Control	Control	Control
Sample size	71,025	71,031	70,951	70,846
R-value squared	0.046	0.065	0.025	0.051
F-value	202.226	288.164	107.956	223.205

We chose whether the participant experienced illness in the last two weeks as the health shock measuring indicator; Standard errors in parentheses; *p < 0.1, **p < 0.05, ***p < 0.01.

Ordinary Least Square (OLS) focuses on the average amount of household consumption. However, obtaining a comprehensive view of the overall conditional distribution is challenging and prone to the influence of extreme values in the sample data. To address this, we employed quantile regression, which minimizes a weighted mean of the residuals, providing more robust outcomes.

Based on Koenker's work (27), this paper constructed a simplified panel quantile regression analysis of health shocks with three tertiles of 0.25, 0.5, and 0.75. The Min-max normalization method was chosen to process sample data and calculate according to the equation below.


$$y_{i}=\frac{x_{i}-\min\left(x_{i}\right)}{\max\left(x_{i}\right)-\min\left(x_{i}\right)}$$

This model investigated the differential impact of health shocks on the allocation of household expenditure across various quantiles, with results displayed in Table 5. The correlation measure for health shocks on the proportion of health and food spending was always significantly positive. Furthermore, the effects of health shocks on total household consumption varied across different quantiles. Specifically, the influence on increasing the proportion of household medical spending and decreasing the proportion of food expenditure intensified with higher quantiles. This phenomenon is probably because families in higher tertiles suffer more significant health shocks and bear additional medical expenses.

**Table 5:** Quantile regression.

	MedExpend %			FoodExpend %		
	*q* = 0.25	*q* = 0.5	*q* = 0.75	*q* = 0.25	*q* = 0.5	*q* = 0.75
Health shock	0.042*** (0.0012)	0.106*** (0.0025)	0.223*** (0.0061)	−0.080*** (0.0073)	−0.123*** (0.0084)	−0.153*** (0.0097)
Control variables	Control	Control	Control	Control	Control	Control

Our paper sets three quantiles for health shocks to analyze the changes in the consumption structure of households affected by different intensities. Standard errors in parentheses. *p < 0.1, **p < 0.05, ***p < 0.01.

The analysis also examined heterogeneity based on gender and income status. Based on the price levels of the corresponding year, the current poverty threshold in 2020 is close to 4,000 yuan. Therefore, we will use this data as the criterion for sample grouping. Table 6 offers an analysis segregated by gender and income to examine altered consumption structure in various gender and income groups following health shocks.

**Table 6:** Heterogeneity analysis 1.

	Male				Female			
	MedExpend %	FoodExpend %			MedExpend %		FoodExpend %	
	Income <¥4,000	Income >¥4,000	Income <¥4,000	Income >¥4,000	Income <¥4,000	Income >¥4,000	Income <¥4,000	Income >¥4,000
Health shock	2.183*** (0.1069)	1.315*** (0.0757)	−1.060*** (0.1459)	−0.467*** (0.1161)	2.335*** (0.0765)	1.351*** (0.0881)	−0.986*** (0.1030)	−0.282** (0.1328)
Control variable	Control	Control	Control	Control	Control	Control	Control	Control
Sample size	9,486	6,839	9,489	6,838	17,167	4,690	17,161	4,691
R-value squared	0.082	0.062	0.104	0.061	0.088	0.057	0.083	0.064
F-value	56.154	30.017	73.357	29.742	110.253	18.865	103.720	21.380

Based on the price levels of the corresponding year, the current poverty threshold in 2020 is close to 4,000 yuan. Standard errors in parentheses;*p < 0.1, **p < 0.05, ***p < 0.01.

Findings show that individual consumption strategies were altered by health shocks, with a notable increase in medical spending and a decline in food intake, corroborating earlier conclusions. From an income perspective, the increase in the percentage of medical expenditures for individuals earning below ¥4,000, was more pronounced compared to those earning more. The reason might be that consumption limits low-income groups, where people spend more on inflexible necessities like food and accommodation, intensifying the detrimental impact of health shocks.

From a gender perspective, differences in consumption habits between men and women were not significantly pronounced. Women exhibited a slightly higher shift in the percentage of medical expenses than men. Nevertheless, the variation in food expense percentage is less pronounced than men’s, suggesting that women are more likely to curtail costs beyond medical and food expenditures to manage health shocks.

Subsequently, this paper examined the heterogeneity of health insurance and the age structure of households. The median age of households is 40, which served as the boundary for sample division. Additionally, the heterogeneity analysis method Zhao (6) proposed was employed to categorize the sample based on household involvement in health insurance. Findings from the regression analysis are presented in Table 7.

**Table 7:** Heterogeneity analysis 2.

	Purchasing family health insurance				No family health insurance			
	MedExpend %		FoodExpend %		MedExpend %		FoodExpend %	
	Age < 40	Age > 40	Age < 40	Age > 40	Age < 40	Age > 40	Age < 40	Age > 40
Health shock	1.321***	2.160***	−0.428***	−0.889***	0.629***	1.573***	−0.411	−1.021***
	(0.0791)	(0.0553)	(0.1169)	(0.0774)	(0.1927)	(0.2015)	(0.2786)	(0.2724)
Control variable	Control	Control	Control	Control	Control	Control	Control	Control
Sample size	10,177	23,372	10,178	23,371	1,634	1996	1,633	1996
R-squared	0.072	0.100	0.068	0.082	0.055	0.076	0.068	0.077
F-value	56.176	185.382	53.145	149.416	6.717	11.577	8.411	11.885

Standard errors in parentheses; *p < 0.1, **p < 0.05, ***p < 0.01.

The trend of the growing proportion of medical spending and a decrease in the percentage of food intake aligned with the principal regression findings. Higher-aged individuals facing health shocks showed a quicker rise in the percentage of medical spending and a more significant drop in the percentage of food expenditure. The data indicates that individuals accumulate health deficits as they age, increasing the need for medical spending to manage health shocks. Data analysis concerning health insurance reveals that the share of medical expenditures grew more rapidly for the sample group that purchases health insurance. There was no significant difference in the share of food expenditures. This phenomenon may be attributed to adverse selection (4) in health insurance enrollment, where individuals with poorer health conditions are more likely to acquire health insurance than those with better health. Even with the mitigating effect of Medicare on health shocks, the proportion of individual health expenditures continues to increase rapidly.

Finally, we analyzed the effect of the urban–rural difference on the structure of consumption. The results of the regression analysis are presented in Table 8. There are significant differences in household consumption structure between urban and rural areas, and the impact of health shocks on rural households is significantly greater Specifically, the proportion of changes in the main expenditures of urban households is smaller than that of rural households when faced with health shocks. The share of changes in welfare expenditure is not obvious and therefore not included in the analysis. This may be because urban areas have a more complete healthcare system than rural areas, and urban households can respond to the adverse effects of health shocks promptly.

**Table 8:** Heterogeneity analysis 3.

	Urban				Country			
	Med %	Food %	Eec %	Epwelf%	Med %	Food %	Eec %	Epwelf%
Health shock	1.606*** (0.0555)	−0.637*** (0.0868)	−0.228*** (0.0606)	−0.032 (0.0295)	2.024*** (0.0634)	−0.855*** (0.0833)	−0.316*** (0.0649)	−0.022 (0.0199)
Control variables	Control	Control	Control	Control	Control	Control	Control	Control
*N*	17,927	17,933	17,895	17,885	20,255	20,246	20,252	20,201
r^2^	0.072	0.048	0.021	0.042	0.079	0.111	0.017	0.048
F	115.230	75.182	31.957	65.089	144.273	209.866	29.290	85.158

Standard errors in parentheses; *p < 0.1, **p < 0.05, ***p < 0.01.

## 6 Research conclusions

Our study employs an empirical methodology to investigate how health shocks impact the consumption structure of Chinese households, utilizing five periods of CFPS panel data from 2010 to 2018. Our empirical analysis yields several conclusions: (1) Health shocks significantly influence household consumption structures. In response to such shocks, households tend to increase the percentage of medical spending significantly while reducing food spending. This strategy aims to mitigate the adverse effects of uncertain health shocks. (2) By incorporating considerations of uncertainty, we regress stochastic medical expenditures as the core explanatory variable. The model estimates are robust and reliable. (3) There is significant income heterogeneity in the effect of health shocks on household consumption structures. Low-income, rural and older individuals experience a more rapid increase in the proportion of medical expenditures following health shocks. (4) Individuals who purchase health insurance tend to increase their proportion of medical expenditures more rapidly following health shocks. This phenomenon may be attributed to adverse selection, where health insurance becomes more attractive to those with poorer health status.

Based on our analysis, our paper presents several suggestions: First, prioritize improving public health. Develop strategies to enhance overall health, reduce the likelihood of health emergencies, and empower individuals to effectively confront and manage health shocks. Secondly, intensify reforms in the medical system. There is a critical need to reduce the impact of medical expenses, alleviate family health pressures, and unlock the potential for household consumption. The last, promote the expansion and improvement of household consumption structures. Increasing disposable income and improving the composition of household expenditures can effectively diminish the impact of health shocks and enhance household well-being.

## Data availability statement

Publicly available datasets were analyzed in this study. This data can be found at: CFPS.

## Author contributions

YQ: Data curation, Formal analysis, Visualization, Writing – original draft. FZ: Conceptualization, Funding acquisition, Methodology, Supervision, Writing – review & editing.
